# Investigation of appropriate needle length considering skin thickness with the real injection posture for insulin injections in diabetic patients

**DOI:** 10.1186/s40780-023-00288-9

**Published:** 2023-06-01

**Authors:** Aya Torii-Goto, Kana Hirai, Yuri Inukai, Yoshimi Hoshina, Kazumi Shiomi, Junko Ito, Masae Yoshikawa

**Affiliations:** 1 Department of Pharmacy, College of Pharmacy, Kinjo Gakuin University, 2-1723 Omori, Moriyama-Ku, Nagoya, 463-8521 Japan; 2 Cure Pharma, 6-1-3 Shimoichiba-Cho, Toyota, 471-0875 Japan; 3 Ito Physiology Clinic, 6-1 Shimoichiba-Cho, Toyota, 471-0875 Japan

**Keywords:** Diabetes, Insulin, Needle, Skin thickness, Injection technique

## Abstract

**Background:**

Insulin treatment is widely used not only for type 1 but also for type 2 diabetes patients. Insulin must be injected into the subcutaneous tissue to be effective. The needle length has been shortened for safety and efficiency. However, whether patients use an appropriate needle length is unclear.

**Methods:**

Skin thickness was measured by ultrasound with patients in their usual posture during injection. Furthermore, the effect of the intervention in which the needle length was changed was investigated.

**Results:**

Thirty-eight percent of the patients had fluid leakage and injected the needle intradermally. The average skin thickness was 3.3 mm while sitting, which was greater than that in a previous report including measurements taken while lying down. Consequently, the skin thickness was > 4 mm in 9.5% of the patients who used 4-mm needles. Cases of leakage and intradermal injection decreased when the needle length was changed.

**Conclusions:**

This study identified that the needle length should be considered in patients with thick skin or a lower body mass index due to possibility of intradermal injection.

## Background

Insulin treatment is widely used not only for type 1 but also for type 2 diabetes patients [1, 2]. Insulin must be injected into the subcutaneous tissue, and not into the muscles, dermis, or nerves [3]. Blood flow through subcutaneous tissue is slow compared to that through muscles [4]. Intramuscular injection can lead to faster absorption than the desirable rate, depending on whether the muscle is at rest or tense [5], causing hypoglycemia [3–5]. Intradermal injection causes hyperglycemia, insulin leakage, and pain [6–8]. Insulin is administered when the needle completely punctures the skin (epidermis and dermis) and enters the fat but does not enter the fascia or muscle [5]. The lengths of the needles are 4, 5, 6, or 8 mm. The most appropriate needle length for patients depends on the skin thickness (ST) and distance to the muscle fascia [3]. Previous reports revealed that the mean ST is 2.20–2.29 mm when lying down via ultrasound [3, 5, 7]. The use of 4-mm needles is widely recognized to be appropriate for all injections regardless of sex, age, and body mass index (BMI) [5]. Because ultrasound measurements are conventionally performed in the lying position, the ST in the usual posture during injection is unclear.

This study measured ST in the usual posture during injection using ultrasound, and problems with injection were investigated in diabetes patients using 4-mm needles to determine the most appropriate needle length. Moreover, this study investigated the effect of the intervention in which the needle length was changed.

## Methods

### Study population

This was a prospective observational study with diabetes outpatients who had received insulin therapy and started using 4-mm needles at Ito Physical Clinic in Toyota, Japan between 27 February and 31 March 2018. Moreover, Japanese patients with type 1 or 2 diabetes were included in this study. Demographic characteristics, including age, sex, weight, BMI, and HbA1c level (NGSP), were obtained from electronic records. Any potentially eligible patient from the clinic outpatients was invited to participate in the study. The major exclusion criteria for this study were an age less than 18 years, unstable diabetes mellitus with repeated hypoglycemia or hyperglycemia, pregnancy, skin disorders, and cancer. Certified diabetes educators checked the injection technique using an evaluation sheet (Table 1) referenced from the Japanese diabetes educators’ guidebook [9]. Patients were evaluated regarding elements of their injection technique by the evaluation sheet. Patients who could not complete any of the elements included in the evaluation sheet were excluded. This study was approved by the ethics committee of Kinjo Gakuin University Pharmaceutical Ethics Committee and performed following the Good Clinical Practice Guidelines (approval number: H18018).

**Table 1:** Injection evaluation sheet

Rolling cloudy (In case of drug mixtures)
Positioning the needle along the axis
Removal of air
Setting the dose
Pressing the dose button
Holding the pen
Removing the needle
Discardment

### Measurement of skin thickness and subcutaneous adipose layer thickness

**Fig. 1:**
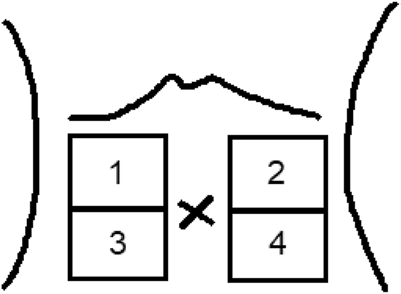
Measurement site of skin thickness on the abdomen

The ST and subcutaneous adipose layer thickness (SCT) were determined based on the total thicknesses of the epidermis and dermis as well as the subcutaneous tissue thickness [3]. ST and SCT were measured using ultrasound equipment (HONDA ELECTRONICS HS-1500, Honda, Japan). The patients were placed in their usual position when performing injections. The medical staff of the clinic measured the patient's ST and SCT. The ultrasound was calibrated for normality. Gel was applied to the ultrasound probe at a right angle above the desired location using the location chart created by Becton, Dickinson and Company (Texas, USA). Abdominal injection sites were indicated by a total of four points using a site chart as described in Fig. 1. The greatest thickness at four points on the abdomen was analyzed to evaluate whether a shorter 4-mm needle was appropriate for patients undergoing site rotation. Moreover, ST and SCT were measured by using 7.5 MHz as the basic frequency. Thickness was measured by the automated machine after the point where each constant thickness was identified by scanning within the range of the desired location.

### Intervention

The needle length was changed from 4 to 6 mm when the ST was 2.9 mm or greater. When injections are performed with 4-mm needles at an angle of 45 degrees, intradermal injections occur at 2.8 mm depth from the skin surface. With a 4-mm needle, it is recommended that patients inject at a 90° angle and avoid pinching-up the skin at the injection site to prevent insulin leakage and intradermal injection. This protocol differed from that for a 6-mm needle. The insulin technique produced two metrics: (1) the insertion angle (vertical or tilted) and (2) pinching-up (yes or no). If a change in the needle length necessitated a change in the injection technique, we taught the technique to the patient. The problems related to injections using 4-mm needles produced two metrics: (1) insulin leakage (yes or no) and (2) intradermal injection (yes or no). A certified diabetes educator scheduled the next (second) visit within less than 3 months after the first visit.

### Statistical analyses

ST and SCT are expressed in millimeters. The characteristics of the patients and their answers to the questionnaire are reported as a percentage of the number of patients. The relationships between two parameters were determined using Spearman's rank correlation coefficient. Analysis of HbA1c changes before and after the intervention was performed using the Mann–Whitney *U* test. Analysis of changes in injection techniques was performed using the chi-square test. *P* < 0.05 was considered statistically significant.

## Results

### The clinical characteristics of the patients

There were 26 eligible patients who had received insulin therapy between 27 February and 31 March 2018. Four patients were excluded for using 6-mm needles. A patient who had not done “Removal of air” in the evaluation sheet were excluded. Thus, this study was conducted with 21 subjects (81%). The clinical characteristics of the 21 patients are shown in Table 2. The mean BMI and HbA1c level were 25.7 ± 8.7 kg/m^2^ and 7.3 ± 0.8%, respectively. The patients had type 1 (four patients, 19%) or type 2 (17 patients, 81%) diabetes. All patients performed injections in the abdomen while sitting in a chair, and pen-type 4-mm needles were used. A total of 86% and 14% of the patients performed injections in the vertical and tilted positions, respectively. Eighty-one percent of the patients pinched-up the skin. Consequently, 38% of the patients had insulin leakage and injected the needle intradermally.

**Table 2:** Background of patients

Patients	21
Gender	
Male	7(33%)
Female	14(67%)
Age (years)	56.5 ± 16.8
Weight (kg)	62.8 ± 16.7
BMI (kg/m^2^)	25.7 ± 8.7
HbA1c (%)	7.3 ± 0.8
Type of diabetes	
Type 1	4(19%)
Type 2	17(81%)

Values in age, weight, BMI, and HbA1c indicated mean ± SD. The other indicated number (percentage) of patients

### Skin thickness and subcutaneous adipose layer thickness

The mean ST and SCT were 3.3 ± 0.6 mm (range, 1.9–4.3 mm) and 16.1 ± 8.7 mm (range, 5.8–44.4 mm), respectively, in the usual posture during injection. Two patients (9.5%) had ST > 4 mm.

### The relationship between intradermal thickness and BMI

**Fig. 2:**
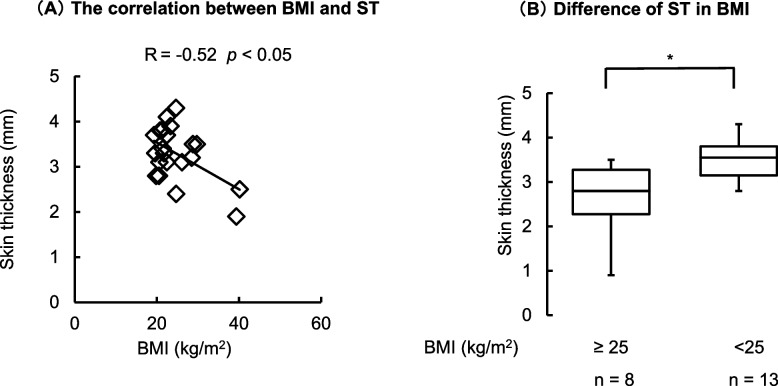
The relationship between BMI and skin thickness. **A** The correlation between BMI and ST (*n* = 21). *P* < 0.05, * P < 0.05 (Spearman coefficient index). **B** Difference in ST based on BMI. The figure indicates median and ranges. (*n* = 21). * P < 0.05 vs. ≥ 25% (Mann–Whitney U test). BMI body mass index

ST was related to BMI (*P* < 0.05; Fig. 2A). Patients with a BMI ≥ 25 kg/m^2^ had a significantly thinner ST (*P* < 0.05; Fig. 2B).

### The relationship between the injection technique and skin thickness

**Fig. 3:**
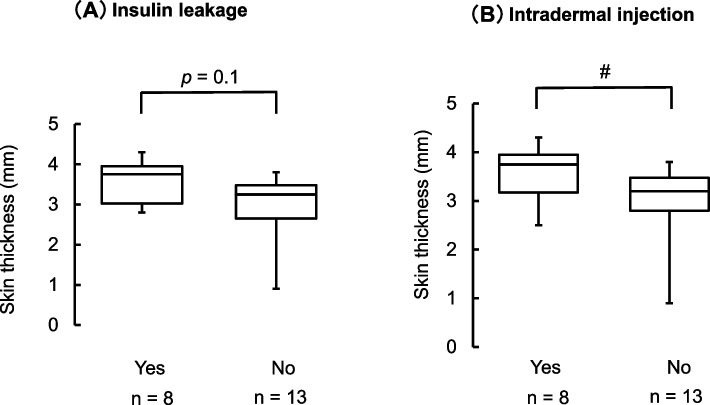
The relationship between injection techniques and skin thickness. The figures indicate the median and ranges (*n* = 21). **A** Insulin leakage. *p* = 0.1. **B** Intradermal injection. *P* < 0.1, #* P* < 0.1 vs. yes (Mann–Whitney U test)

The results of the questionnaire are shown in Fig. 3. Patients without intradermal injection tended to have a high ST (*P* = 0.07; Fig. 3B). ST was not associated with insulin leakage (*P* = 0.1; Fig. 3A).

### The effect of changing the needle length and injection technique interventions

**Fig. 4:**
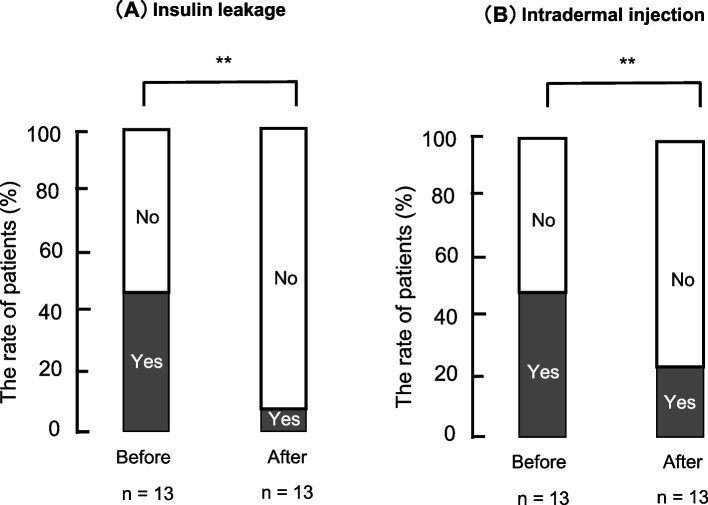
The effect of the intervention injection technique and skin thickness. **A** Insulin leakage. **B** Intradermal injection. Values indicate the rate of yes or no answers (*n* = 13). *P* < 0.01, ** P < 0.01 vs. yes (chi-square test)

The needle length and injection technique were changed for 71% (*n* = 15) and 14% (*n* = 3) of the patients, respectively. The results of the interventions are shown in Fig. 4. HbA1c and insulin requirements did not change before or after the intervention. However, insulin leakage and intradermal injection significantly decreased from 46% (*n* = 6) to 8% (*n* = 1) and from 46% (*n* = 6) to 23% (*n* = 3), respectively (*P* < 0.01; Fig. 4A, B).

## Discussion

The recommended needle lengths for insulin injection have been changing for a long time. Although almost half of the patients used 8-mm needles until 2010 [10], 4-mm needles were common at the time of study [11]. Shorter needles are less likely to put patients at risk for an intradermal injection [8, 12, 13]. Shorter needles are less painful, easier to use, and more favorable for adult Japanese diabetes patients [14–16]. Gibney et al. reported that an insertion angle of 45° with a 4-mm needle increases the risk of intradermal injections by 7.7% [7]. In this study, more patients (38%) had fluid leakage and injected the needle intradermally although subjects with poor injection techniques were excluded. It was hypothesized that a 4-mm needle might not be appropriate for some patients. Thus, we focused on ST.

Consideration of ST is important for subcutaneous tissue injections to avoid injection site pain and uncontrolled blood glucose [5]. The average ST was shown to be 2.20–2.29 mm across several races [7] and in Asians [3, 17, 18] when the usual measurement method was used. Many diabetes patients now use shorter needles, especially 4-mm needles, regardless of their BMI. However, the ST in the actual posture during injection (e.g., while sitting) is unclear. Although a shorter needle length is the standard, diabetes educators should assess its appropriateness and effectiveness on a case-by-case basis and should recommend longer needles when needed [19].

This study investigated ST in the usual posture while lying down. The average ST when sitting was 3.3 mm, which was greater than that while lying down. The average SCT in this study was 16.1 mm. However, another study showed that it was 10.15–13.92 mm [3, 7]. The difference in posture was suggested to be related. The ST was > 4 mm in 9.5% of the patients. Thus, injection into the subcutaneous tissue with vertical puncture was not performed. Previous reports mentioned that ST increased with increasing BMI [3, 7]. However, this study confirmed that the higher the BMI, the lower the ST. It is suggested that ST is increased when measured in the actual posture during injection. Attention should be given to fluid leakage and intradermal injection in patients with lower BMI. Additionally, the needle should be changed to a longer needle in patients with lower BMI. The number of subjects in this small study necessitates further investigation.

In this study, the needle length was changed to 6-mm based on the ST results. Leakage and intradermal injection decreased after the intervention. The average ST did not change with any needle length [20]. Partial or full remission of tissue swelling due to insulin injection can take up to one year [21]. Injection-related problems may be reduced by changing the needle length in a custom-made manner according to each patient's ST. However, ST needs to be measured after intervention. The proper selection of needles and professional education can result in improved insulin injection techniques, higher patient satisfaction, and better glycemic control [22, 23]. No changes in insulin dosage or HbA1c levels were observed in this study. The patients’ STs were not compared via ultrasound while lying down and sitting. Further analyses are needed to determine the effect of glycemic control using different interventions with larger sample sizes.

## Conclusions

This study revealed that needle length should be considered in cases of high ST or low BMI because of the risk of intradermal injection. These findings support the idea of establishing individualized supportive therapies for diabetes patients. This study theoretically contributes to increased safety of insulin injection therapy and greater quality of life for patients.

## Data Availability

All data generated or analyzed during this study are included in this published article.

## Abbreviations

IM: Intramuscular
BMI: Body mass index
ST: Skin thickness
HbA1c: Hemoglobin A1c
NGSP: National Glycohemoglobin Standardization Program
SCT: Subcutaneous adipose layer thickness

## References

[1] Lefever E, Vliebergh J, Mathieu C (2021). Improving the treatment of patients with diabetes using insulin analogues: current findings and future directions. Expert Opin Drug Saf.

[2] Silver B, Ramaiya K, Andrew SB, Fredrick O, Bajaj S, Kalra S (2018). EADSG guidelines: insulin therapy in diabetes. Diabetes Ther.

[3] Sim KH, Hwang MS, Kim SY, Lee HM, Chang JY, Lee MK (2014). The appropriateness of the length of insulin needles based on determination of skin and subcutaneous fat thickness in the abdomen and upper arm in patients with type 2 diabetes. Diabetes Metab J.

[4] Lim STJ, Hui YCA, Lim PK, Lim CCE, Yen Chia Y, Vasanwala RF (2018). Ultrasound-guided measurement of skin and subcutaneous tissue thickness in children with diabetes and recommendations for giving insulin injections. J Clin Transl Endocrinol.

[5] Hirsch LJ, Strauss KW (2019). The injection technique factor: what you don't know or teach can make a difference. Clin Diabetes.

[6] Down S, Kirkland F (2012). Injection technique in insulin therapy. Nurs Times.

[7] Gibney MA, Arce CH, Byron KJ, Hirsch LJ (2010). Skin and subcutaneous adipose layer thickness in adults with diabetes at sites used for insulin injections; implications for needle length recommendations. Curr Med Res Opin.

[8] Lippert WC, Wall EJ (2008). Optimal intramuscular needle-penetration depth. Pediatrics.

[9] Certification board for diabetes educators in Japan (2021). Guidebook for diabetes educate in 2021.

[10] De Coninck C, Frid A, Gaspar R, Hicks D, Hirsch L, Kreugel G (2010). Results and analysis of the 2008–2009 Insulin Injection Technique Questionnaire survey. J Diabetes.

[11] Hirsch L, Klaff L, Bailey T, Gibney M, Albanese J, Qu S (2010). Comparative glycemic control, safety and patient ratings for a new 4 mm × 32G insulin pen needle in adults with diabetes. Curr Med Res Opin.

[12] Hirsch L, Byron K, Gibney M (2014). Intramuscular risk at insulin injection sites: measurement of the distance from skin to muscle and rationale for shorter-length needles for subcutaneous insulin therapy. Diabetes Technol Ther.

[13] Kreugel G, Keers JC, Kerstens MN, Wolffenbuttel BH (2011). Randomized trial on the influence of the length of two insulin pen needles on glycemic control and patient preference in obese patients with diabetes. Diabetes Technol Ther.

[14] Miwa T, Itoh R, Kobayashi T, Tanabe T, Shikuma J, Takahashi T (2014). Comparison of the effects of a new 32-gauge × 4-mm pen needle and a 32-gauge × 6-mm pen needle on glycemic control, safety, and patient ratings in Japanese adults with diabetes. Diabetes Technol Ther.

[15] Nagai Y, Ohshige T, Arai K, Kobayashi H, Sada Y, Ohmori S (2013). Comparison between shorter straight and thinner microtapered insulin injection needles. Diabetes Technol Ther.

[16] Birkebaek NH, Solvig J, Hansen B, Jorgensen C, Smedegaard J, Christiansen JS (2008). A 4-mm needle reduces the risk of intramuscular injections without increasing backflow to skin surface in lean diabetic children and adults. Diabetes Care.

[17] Wang W, Guo X, Shen G, Bai G, Wei Z, Liu J (2016). Skin and subcutaneous tissue thickness at insulin injection sites in Chinese diabetes patients: clinical implications. Diabetes Metabo.

[18] Jain SM, Pandey K, Lahoti A, Rao PK (2013). Evaluation of skin and subcutaneous tissue thickness at insulin injection sites in Indian, insulin naïve, type-2 diabetic adult population. Indian J Endocrinol Metab.

[19] Hansen B, Matytsina I (2011). Insulin administration: selecting the appropriate needle and individualizing the injection technique. Expert Opin Drug Deliv.

[20] Jung HJ, Kim DW, Chung SL, Kim TH (1990). A study on the skin thickness of Koreans by ultrasound. Korean J Dermatol.

[21] Hauner H, Stockamp B, Haastert B (1996). Prevalence of lipohypertrophy in insulin-treated diabetic patients and predisposing factors. Exp Clin Endocrinol Diabetes.

[22] Gorska-Ciebiada M, Masierek M, Ciebiada M (2020). Improved insulin injection technique, treatment satisfaction and glycemic control: results from a large cohort education study. J Clin Transl Endocrinol.

[23] Nakatani Y, Matsumura M, Monden T, Aso Y, Nakamoto T (2013). Improvement of glycemic control by re-education in insulin technique in patients with diabetes mellitus. Adv Ther.

